# The quest for the holy grail: new antitubercular chemical entities, targets and strategies

**DOI:** 10.1016/j.drudis.2020.02.003

**Published:** 2020-04

**Authors:** Stanislav Huszár, Kelly Chibale, Vinayak Singh

**Affiliations:** 1 Drug Discovery and Development Centre (H3D), University of Cape Town, Rondebosch 7701, South Africa; 2 South African Medical Research Council Drug Discovery and Development Research Unit, Department of Chemistry and Institute of Infectious Disease and Molecular Medicine, University of Cape Town, Rondebosch 7701, South Africa

## Abstract

Tuberculosis (TB) remains the leading cause of death from an infectious disease worldwide. TB therapy is complicated by the protracted treatment regimens, development of resistance coupled with toxicity and insufficient sterilizing capacity of current drugs. Although considerable progress has been made on establishing a TB drug pipeline, the high attrition rate reinforces the need to continually replenish the pipeline with high-quality leads that act through inhibition of novel targets. In this review, we highlight some of the key advances that have assisted TB drug discovery with novel chemical matter, targets and strategies – to fuel the TB drug pipeline.

## Highlights

• In 2018 1.2 million people died of tuberculosis.
• The ideal drug candidate should be active against replicating and nonreplicating *Mycobacterium tuberculosis*.
• New drug targets such as EfpA, PptT, ClpP, Pks13, DnaN and QcrB have been identified.
• Tuberculosis drug discovery is advancing with innovative screens.

## Introduction

Tuberculosis (TB), caused by *Mycobacterium tuberculosis*, has accompanied mankind throughout history and is still one of the top ten causes of death worldwide. In 2018, 1.2 million people died of TB (of which 251,000 were HIV positive) and 10 million people developed the disease [1]. About 1.7 billion people, 23% of the world’s population, are estimated to have a latent TB infection and are thus at risk of developing active disease during their lifetime. Drug-susceptible (DS) TB (Box 1) is treated for 6 months with a combination of anti-TB drugs: isoniazid, rifampicin, pyrazinamide and ethambutol, which were discovered >60 years ago. However, this global health burden is exacerbated by multidrug-resistant (MDR) and extensively drug-resistant (XDR) TB (Box 1) where the treatment runs up to 2 years with limited success.

Box 1 **Challenges in tuberculosis drug discovery and key definitions**

The goals and challenges for improved tuberculosis (TB) therapy can be listed as: (i) to discover and develop new chemical matter with novel mechanism(s) of action (MoA) – to treat drug-resistant strains and contribute to treatment shortening; (ii) to find novel bactericidal compounds effective against persistent and dormant bacilli – to contribute to treatment-shortening; and (iii) discovering drugs with no drug–drug interactions – for better drug combinations.

*Drug-susceptible tuberculosis (DS-TB)*

TB caused by a *Mycobacterium tuberculosis* strain sensitive to the first-line anti-TB drugs rifampicin and isoniazid.

*Multidrug-resistant TB (MDR-TB)*

TB caused by *M. tuberculosis* strain resistant to at least the two-most-effective first-line anti-TB drugs rifampicin and isoniazid.

*Extensively drug-resistant TB (XDR-TB)*

TB caused by MDR strains that are also resistant to any of the fluoroquinolones and at least one injectable second-line drug.

*Persistent M. tuberculosis*

*N*onreplicating *M. tuberculosis* that can withstand host defence mechanisms and antibiotic treatment without heritable resistance – it usually arises from the hetero-tolerant population of bacteria.

*Granuloma*

An organized collection of macrophages and immune cells, formed as a result of *M. tuberculosis* infection.

*Caseum*

The center of necrotizing granuloma with an extracellular pathogen, resulting from necrotic lysis of host immune cells.

*Foamy macrophage*

A lipid-rich pulmonary macrophage formed as a result of *M. tuberculosis* infection. At a later stage, it can necrotize to release intracellular *M. tuberculosis* and free accumulated lipid droplets, which then form a cheese-like substance of the caseum.

*CRISPR interference (CRISPRi)*

Molecular-engineered and scalable platform for regulated gene silencing in mycobacteria based on the bacterial CRISPR-Cas system (CRISPR, clustered regularly interspersed short palindromic repeats) utilizing sgRNA and dCas9.

*sgRNA*

Single guide RNA, designed to specifically bind to the target DNA region making the DNA/RNA hybrid.

*dCas9*

Catalytically inactive (also called dead) nuclease Cas9 from *Streptococcus thermophilus* (dCas9Sth1), which forms a complex with sgRNA, thereby blocking the gene transcription by RNA polymerase.

Alt-text: Box 1

One of the luxuries of *M. tuberculosis*, which makes it difficult to combat by antibiotics, is its highly hydrophobic and less permeable cell envelope. Another advantage that qualifies it as a successful intracellular pathogen is the ability to outwit the host’s immune system and persist within the alveolar macrophage in a hypometabolic nonreplicating form. The main difficulty of TB therapy is thus to target and kill the drug-tolerant and persistent *M. tuberculosis* (Box 1) residing in the TB lesions 2, 3. Therefore, to eradicate *M. tuberculosis* from the host tissues and to prevent the relapse of TB, there is a need to identify novel anti-TB drugs targeting replicating and nonreplicating stages of the bacillus.

TB drug discovery and development has made great progress in the past 20 years in part owing to the deciphering of the *M. tuberculosis* genome in 1998 [4]. Coupled with methods of identifying essential genes, this has facilitated the understanding of crucial biochemical processes, which can be targeted to treat TB. In this review, we highlight the latest discoveries of new chemical entities, drug targets and screening strategies that can help to supplement the current TB drug pipeline and accelerate TB drug discovery and development.

## Attractive chemical matter: need of the hour

The most advanced anti-TB drug candidates from the current drug pipeline (https://www.newtbdrugs.org/pipeline/clinical) target energy metabolism through inhibition of ATP synthase and respiratory cytochrome *bc*_1_ complex or cell-wall biosynthesis.

### Promising compounds in preclinical development

**Figure 1:**
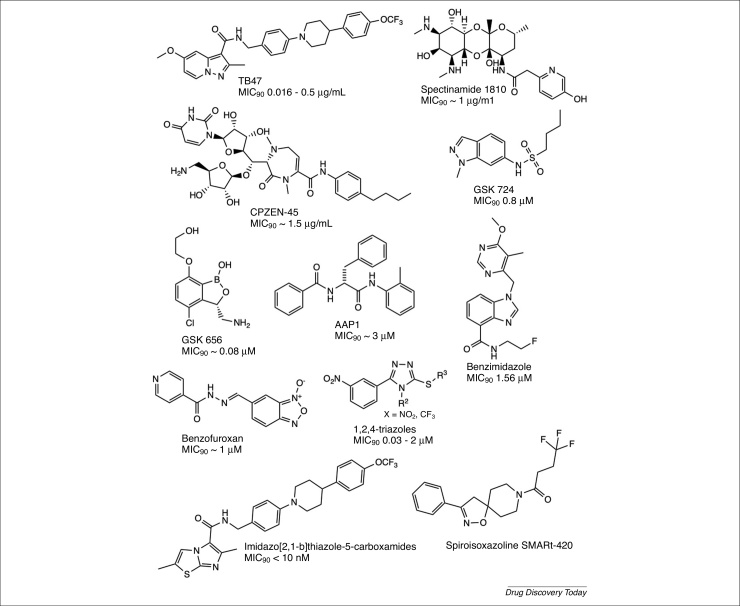
Novel antitubercular compounds. Chemical structures of the promising compounds in preclinical development (TB47, spectinamide 1810 and CPZEN-45) and novel chemical scaffolds (GSK656, AAP1, benzimidazole, 1,2,4-triazole, benzofuroxan, imidazol[2,1-b]thiazole-5-carboxamides and SMARt-420) with their MIC_90_ values against *Mycobacterium tuberculosis* H37Rv strain, where applicable.

TB47, a pyrazolo[1,5-a]pyridine-3-carboxamide hybrid (Fig. 1), was developed using a scaffold-hopping strategy to improve druggability and potency [5]. It is structurally similar to imidazopyridine amide compound Q203 and targets the QcrB subunit of respiratory cytochrome *bc_1_* complex [6]. TB47 was equally active against DS and MDR/XDR clinical isolates, exhibited good selectivity index of 1200 to >3330, displayed no activity on cytochrome P450 (up to 20 μM) and good oral bioavailability of 94.3% with a half-life of 19.1 ± 3.2 h. The lack of toxicity in human cell lines and rat models, combined with high oral bioavailability in rats, favors TB47 as an oral medication [7].

Spectinamides are novel semisynthetic derivatives of spectinomycin with a narrow-spectrum anti-TB activity, active under hypoxic nonreplicating conditions, lacking cross-resistance to existing anti-TB drugs and exhibiting an excellent pharmacological profile (Fig. 1) [8]. The high ribosomal affinity and ability to overcome Rv1258c-mediated efflux enabled the improved activity. Although spectinomycin is chemically similar to aminoglycosides, it binds to a different site within the 16S ribosomal subunit ‘helix 34’ and blocks ribosome translocation. Robertson *et al.* [9] have shown that the spectinamides are effective in multiple murine TB models and, most importantly, showed a significant synergy with the existing anti-TB drugs.

CPZEN-45 is a semisynthetic derivative of the natural product caprazamycin and is active against DS (1.56 μg/ml) and MDR (6.25 μg/ml) *M. tuberculosis* (Fig. 1). It showed excellent therapeutic efficacy in a murine TB model infected with an XDR strain resistant to ten drugs [10]. It targets WecA/Rv1302 and inhibits the first step of arabinogalactan biosynthesis, which was recently validated as a potential drug target using biochemical and genetic approaches [11]. A recent study combines CPZEN-45 with capreomycin as components of single particles by spray-drying, yielding a new aerosol combination drug therapy for MDR-TB and XDR-TB – the pharmacokinetics showed high local concentrations of CPZEN-45 and capreomycin following direct administration to the lungs and subsequent systemic bioavailability [12].

### Novel chemical scaffolds

Rifampicin, a first-line anti-TB drug, has for many years been an integral part of TB chemotherapy. However, the expansion of rifampicin-resistant strains impedes its use. Lin *et al.* [13] discovered non-rifampicin-related compounds: Na-aroyl-*N*-aryl-phenylalaninamides (AAPs) that potently and selectively inhibit *M. tuberculosis* RNA polymerase (*Mtb*RNAP) and mycobacterial growth (Fig. 1). Crystal structures of *Mtb*RNAP in complex with AAPs revealed that AAPs bind to a different site where rifampicin does not bind, exhibited no cross-resistance, showed an additive effect and suppressed the mutation frequency when co-administered with rifampicin.

Benzimidazoles were identified as noncovalent DprE1 inhibitors with good efficacy in a murine model of TB (Fig. 1) [14]. Molecular modeling of the benzimidazoles suggested plausible modes of binding in the active site of DprE1. The 3,5-dinitrophenyl 1,2,4-triazoles are also novel DprE1 inhibitors (Fig. 1) [15]. SAR studies revealed that the 3,5-dinitrophenyl fragment is crucial for the anti-TB activity. S-substituted 4-alkyl-5-(3,5-dinitrophenyl)-4H-1,2,4-triazole-3-thiols and their 3-nitro-5-(trifluoromethyl)phenyl analogs showed the best activity against MDR clinical isolates.

The indazole sulfonamide GSK3011724A belongs to another novel class of small molecules identified from phenotypic screening with an MIC value of 0.8 μM against DS *M. tuberculosis* (Fig. 1). Analysis of spontaneous-resistant mutants identified several mutations in the essential β-ketoacyl synthase (*kasA*/Rv2245) gene, which was previously reported as a drug target of thiolactomycin [16]. Subsequently, KasA was confirmed as the target of indazole sulfonamides using biochemical assays, chemical proteomics and structural resolution of KasA in complex with GSK3011724A. GSK3011724A is in the lead optimization phase, further validating KasA as a chemically and genetically validated drug target [17].

GSK656 belongs to a novel class of oxaboroles and inhibits Leucyl-tRNA synthetase (LeuRS) (Fig. 1). It displayed good *in vitro* potency as well as excellent *in vivo* efficacy in acute and chronic mouse models [18]. Benzofuroxan, a novel *N*-oxide-containing compound (Fig. 1), is active against replicating and nonreplicating *M. tuberculosis* with an *in vivo* sterilizing effect [19]. A favorable profile for cytotoxicity, safety, absorption, distribution and metabolism supports the compound as a promising lead for further development. Initial mechanism of action (MoA) studies suggest that benzofuroxan targets translation. Moraski *et al.* [20] extended the SAR studies of imidazo[1,2-a]pyridine-3-carboxamides, targeting QcrB, and developed a novel class of anti-TB compounds: imidazo[2,1-b]thiazole-5-carboxamides, active against susceptible and resistant *M. tuberculosis*. The most potent compounds from this series showed an MIC of <10 nM.

A potential mechanism for reversing drug resistance in TB can be the induction of alternative bioactivation pathways of anti-TB prodrugs. Ethionamide (ETH), an anti-TB prodrug, is activated by monooxygenase EthA and the active metabolite targets the enoyl reductase (InhA) – inhibiting the mycolic acids biosynthesis 21, 22. EthA overexpression is under the control of TetR-type transcriptional repressor EthR [23], thus the small molecules that bind to the EthR can stimulate EthA expression and boost ETH potency [24]. In this context, Blondiaux *et al.* [25] describe an interesting molecule: SMARt-420, from the spiroisoxazoline class of small molecules aborting resistance (SMARt). SMARt-420 not only stimulates ETH activity but reverts EthA-mediated resistance because it acts via the newly identified *ethR2*-*ethA2* region. A combination of ETH and SMARt-420 showed an impressive reduction in *M. tuberculosis* infection in the lungs of treated mice.

## Novel anti-TB drug targets: opportunities for overcoming resistance

**Figure 2:**
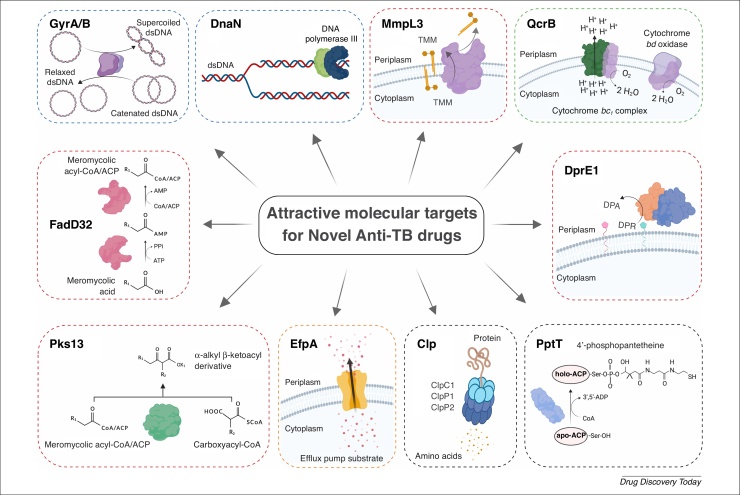
Attractive drug targets. Molecular targets that have been genetically and chemically validated in recent years, with significant progress in their biochemical characterization. The chemical validation is supported by many active inhibitors or at least one active compound. The presented molecular targets have not been targeted by any clinically used first-line anti-TB drugs, making them suitable for targeting MDR or XDR *Mycobacterium tuberculosis* strains. Impairment of their functions leads to the disrupted: (i) nucleic acid synthesis by targeting DNA gyrase – GyrA/B or DNA polymerase III sliding clamp – DnaN (shown by blue dashed line); (ii) cell wall biosynthesis by targeting mycobacterial membrane protein large 3 – MmpL3, decaprenylphosphoryl-β-d-ribose 2′-oxidase – DprE1, fatty acyl-AMP ligase – FadD32 or polyketide synthase – Pks13 (indicated by red dashed line); (iii) oxidative phosphorylation by targeting cytochrome *bc*_1_ complex subunit B – QcrB (indicated by green dashed line); (iv) antibiotic efflux by targeting efflux pump – EfpA (indicated by orange dashed line); and (v) intermediate metabolism by targeting caseinolytic protease complex – Clp or phosphopantetheinyl transferase – PptT (indicated by black dashed line). Abbreviations: dsDNA, double-strand DNA; TMM, trehalose monomycolate; DPR, decaprenylphosphoryl-β-d-ribose; DPA, decaprenylphosphoryl-β-d-arabinose, CoA, coenzyme A; ACP, acyl carrier protein. The illustration was created using BioRender.

The discovery of streptomycin in 1944 was the milestone in TB chemotherapy [26]. As a protein synthesis inhibitor, streptomycin binds to the 16S rRNA of the ribosome, making it the first discovered TB drug target. Here, we focus on some attractive drug targets that have been genetically and chemically validated and can be a basis for tackling drug resistance (DR) (Fig. 2).

### GyrA/B

DNA gyrase, a tetrameric enzyme consisting of GyrA/Rv0006 and GyrB/Rv0005 subunits, is the only type-2 topoisomerase in *M. tuberculosis* and a validated target of fluoroquinolones. Fluoroquinolones inhibit DNA gyrase after DNA cleavage, which results in permanent double-strand (ds)DNA breaks and impaired DNA replication. Moxifloxacin kills replicating as well as anaerobic nonreplicating *M. tuberculosis* [27] and is thus expected to demonstrate sterilizing activity *in vivo*. However, the failure of a Phase III trial [28] involving moxifloxacin, for treatment-shortening, is not necessarily a reflection on DNA gyrase as an attractive target. A possible explanation for the failed clinical trial is provided by a study of the spatial distribution of TB drugs in intact lesions, which revealed that moxifloxacin did not accumulate high enough in the caseum (Box 1) of cavities and nodules to kill nonreplicating *M. tuberculosis* [2]. In our opinion, DNA gyrase, as a target, has the potential to contribute to treatment shortening and overcoming the challenges of treating DR-TB with compounds that have improved human pharmacokinetics and caseum penetration.

### QcrB

*M. tuberculosis*, an obligate aerobe, requires oxygen to replicate, thus making electron-transport-chain complexes an attractive targets for inhibiting respiration. Of these, QcrB/Rv2196, first identified as the target of Phase II clinical candidate Q203 [29], has gained significant attention. Because *M. tuberculosis* also carries CydAB (Rv1623/22c), an alternative nonessential cytochrome *bd* oxidase, QcrB inhibitors have limitations in eradicating the nonreplicating *M. tuberculosis* from the host. This is why it is important to combine QcrB inhibitors with inhibitors of the alternative respiratory pathway. Recently, the crystal structure of the purified mycobacterial respiratory supercomplex III-IV was solved by cryo-EM [30]. Newly identified QcrB inhibitors such as morpholino thiophenes [31] and arylvinylpiperazine amides [32] are providing further impetus to explore QcrB as a target. However, a suitable enzyme assay for medium/high-throughput screening of QcrB inhibitors is still needed.

### DnaN

Sliding clamp DnaN/Rv0002, an essential subunit of DNA polymerase III, has been validated as the target of modified griselymicin [33]. This natural product was highly active against replicating *M. tuberculosis* and effective in a mouse model of TB. Also, the bactericidal activity of griselymicin on nonreplicating cultures can be attributed to the possible involvement of DnaN in the DNA repair mechanisms. Despite the discovery of only one chemical scaffold, DnaN has significantly attracted the attention of TB researchers. Efforts are needed to develop suitable assay(s) to facilitate the screening of chemical libraries to identify novel DnaN inhibitors.

### PptT

Phosphopantetheinyl transferase (PptT/Rv2794c), which transfers 4′-phosphopantetheine from coenzyme A to diverse acyl carrier proteins, is essential for *M. tuberculosis* growth [34]. The recent discovery of an amidino-urea compound (8918) that kills *M. tuberculosis* by inhibiting PptT makes this enzyme one of the novel high priority targets [35]. It was interesting to note that even partial inhibition of PptT was toxic to *M. tuberculosis*, probably owing to 4′-phosphopantetheinyl hydrolase (PptH) synergizing with the inhibitor by undoing the PptT reaction. The availability of the crystal structure of PptT [36] should facilitate target-guided TB drug discovery.

### FadD32

Fatty-acyl-AMP ligase, FadD32/Rv3801c, which is required for activation of the long meromycolic chain of mycolic acids – the hallmark of the mycobacterial cell wall – is an essential enzyme for mycobacterial growth. FadD32 has been biochemically characterized and the available crystal structure opens the door for rational drug design [37]. Using a novel high-throughput enzyme assay, five new classes of FadD32 inhibitors, namely benzimidazole, thiopyrimidine, pyrrozole, quinolone and iso-quinolone, were identified [38]. Diarylcoumarins are potent FadD32 inhibitors and are active in mouse models of TB [39].

### Pks13

The Rv3800c gene is essential for growth and encodes the type-I polyketide synthase, Pks13, involved in the final steps of mycolic acid biosynthesis, specifically the Claisen-type condensation of the C_22_-C_26_ fatty acid with the C_50_-C_60_ meromycolic acid – activated by FadD32 – to produce α-alkyl-β-ketoacids, the precursors of mycolic acids [40]. The keto group is then reduced to the hydroxy group, and the mycolate precursor is transferred to trehalose to form trehalose monomycolate (TMM). TMM is then translocated from the cytoplasm to the outer membrane space, where trehalose monomycolate (TDM) is formed. The depletion of TMM, TDM and mycolic acids are, therefore, typical signatures of Pks13 or FadD32 enzyme inhibition. Pks13 is targeted by the recently reported benzofurans [41] and coumestan derivatives [42].

### MmpL3

MmpL3/Rv0206c has been one of the most recently highlighted drug targets. The transport of mycolic acids across the cytoplasmic membrane was unknown for a long time, until adamantylurea AU1235 and 1,2-ethylenediamine SQ109 were almost simultaneously identified as inhibitors of mycobacterial membrane protein large MmpL3, the TMM transporter 43, 44. The chemical inhibition or genetic inactivation of MmpL3 activity results in the accumulation of TMM inside the cells and suppression of TDM and mycolic acid levels, which has lethal consequences for mycobacteria and, thus, classifies MmpL3 as a highly vulnerable target. A diverse range of MmpL3 inhibitors has recently been reported, categorizing this as a highly promiscuous target. In the context of drug specificity, Li *et al.* [45] developed assays for the identification of direct inhibitors of MmpL3.

### DprE1

The FAD-dependent decaprenylphosphoryl-β-d-ribose oxidase (DprE1/Rv3790) is an essential enzyme targeted by benzothiazinones (BTZs) [46]. The piperazino derivative of BTZ, PBTZ169 [47] (now Macozinone) with its exceptional MIC against *M. tuberculosis* (0.19–0.75 ng/ml) and improved pharmacologic properties has entered Phase II human clinical trials. Several structurally diverse chemotypes targeting DprE1 have recently been identified, suggesting DprE1 to be a promiscuous target. However, the vulnerability of DprE1 is attributed to its periplasmic localization in the cell [48].

### Clp

Clp, a caseinolytic protease complex, plays a pivotal part in protein turnover and bacterial proteome homeostasis. It consists of proteolytic subunits ClpP1/Rv2461c and ClpP2/Rv2460c, and the regulatory ATPase subunit ClpC1/Rv3596c. ClpC1 recognizes misfolded or damaged proteins, unfolds them and passes them into a double-ring tetradecameric ClpP1P2 chamber for proteolytic degradation. Since ClpP1P2 and ClpC1 possess unique enzymatic activities, they are considered individual targets. The ClpP1P2 structure facilitated the design and synthesis of selective bortezomib derivatives with reduced activity against the human proteasome [49]. By contrast, the ClpC1 inhibitor rufomycin has been reported to be effective against replicating and nonreplicating mycobacteria [50].

### EfpA

Efflux pumps play an important part in bacterial resistance to antimicrobial drugs – by decreasing intracellular drug concentration. For effective TB therapy, the treatment regimen should include efflux pump inhibitor(s). Johnson *et al.* [51] identified and optimized the efflux pump EfpA/Rv2846c inhibitor BRD-8000, and validated this previously uncharacterized and essential efflux pump as a novel drug target. In addition to the targets described above, biotin protein ligase, β-ketoacyl-ACP-synthase-I, tryptophan synthase and Leucyl-tRNA synthetase have attracted attention recently.

## Strategies: innovation at its best

**Figure 3:**
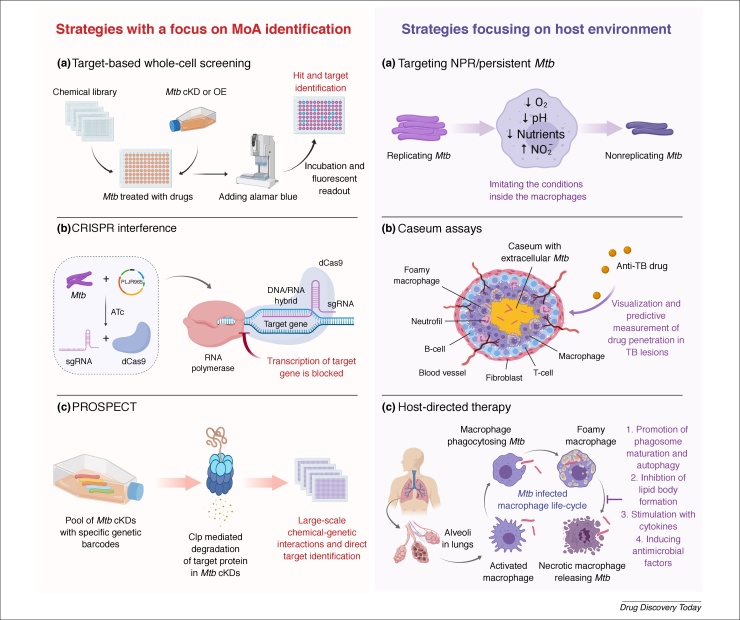
Novel strategies in anti-TB drug discovery. Highlighting some of the new strategies and techniques that can be used to combat tuberculosis (TB). Target-based whole-cell screening, CRISPR interference and PROSPECT utilize the recombinant *Mycobacterium tuberculosis* strains (OEs or cKDs), enabling identification or confirmation of the molecular target of the hit compound from whole-cell screening. Targeting nonreplicating and/or persistent *M. tuberculosis*, Caseum assays and host-directed therapy are focusing on host environment during the *M. tuberculosis* infection. Abbreviations: cKD, conditional knockdown; OE, overexpressor; CRISPR, clustered regularly interspersed short palindromic repeats; ATc, anhydrotetracycline; sgRNA, single guide RNA; dCas9, catalytically inactive (dead) nuclease Cas9; *Mtb*, *M. tuberculosis*; PROSPECT, primary screening of strains to prioritize expanded chemistry and targets; NRP, nonreplicating. The definition of foamy macrophage is described in Box 1. The illustration was created using BioRender.

In search of new anti-TB agents, testing of individual molecules or chemical libraries inhibiting *M. tuberculosis* growth under *in vitro* conditions, phenotypic (whole-cell) screening, has become standard practice. An integral part of many preclinical tests, which follow the discovery of an active hit, is to elucidate the MoA in *M. tuberculosis*. If the original hit does not have appropriate pharmacokinetic and pharmacodynamic properties, then SAR studies and/or rational drug design play a significant part in finding drug-like molecules. By contrast, in traditional target-based screening – the preferred screening approach of pharma companies – compounds are tested directly on the purified target to measure inhibition of enzymatic activity followed by testing of the active hits for whole-cell activity. Here, the principal criteria for selecting a particular target are: (i) vulnerability of the target *in vitro*, *ex vivo* and *in vivo*; and (ii) target selectivity – the lack of a human counterpart. Interestingly, target-based screening has largely not been successful in TB drug discovery owing to: (i) poor compound penetration across the highly impermeable *M. tuberculosis* cell wall; (ii) compound efflux out of the cell; or (iii) compound metabolism inside the cell to an inactive form. These obstacles can sometimes be difficult to overcome even after comprehensive medicinal chemistry efforts. However, the utility of target-based approaches cannot be ignored as evidenced by the discovery of BioA inhibitors involved in *M. tuberculosis* biotin biosynthesis [52]. It is noteworthy that, in recent times, significant efforts have been made in developing improved drug screening tools (Fig. 3).

### Target-based whole-cell screening

MoA of hits from phenotypic whole-cell screening is often difficult and time consuming. Therefore, a more sophisticated approach that combines phenotypic and target-based screening was applied in TB drug discovery. The so-called target-based whole-cell screening utilizes genetically modified strains of *M. tuberculosis* (hypomorphs and overexpressors) in which the target gene and protein expression can be controlled. This allows addressing the on-target activity of the compounds, which is demonstrated by MIC shift: the hypomorphs, depleted in a particular target, show hypersensitivity to the target compound; and the overexpressors, overexpressing the target, become less sensitive to the compound. In parallel, the use of hypomorphs has proven to be very useful in our hands for MoA validation of potential compounds 11, 53, 54. Moreira *et al.* [55] developed a specific type of approach that they named target-mechanism-based whole-cell screening. In this approach, instead of depleting or overexpressing the target protein: ClpP1P2, they engineered a *Mycobacterium smegmatis* strain that overexpresses the GFP-SsrA – the substrate of ClpP1P2. Inhibitors of ClpP1P2 block the degradation of GFP-SsrA, which results in a gain of fluorescent signals. Through this approach, bortezomib was discovered as an inhibitor of the mycobacterial ClpP1P2 proteolytic complex.

### CRISPR interference

The game-changer CRISPR-Cas technology has revolutionized many areas of biomedical research, and TB research is a recent add-on (Box 1). Rock *et al.* [56] customized the CRISPR-Cas system for *M. tuberculosis*. The CRISPR interference (CRISPRi) (Box 1) in mycobacteria utilizes a single plasmid that expresses a mycobacterial codon-optimized nuclease-dead dCas9 under the control of an anhydrotetracycline (ATc)-inducible promoter and the cognate sgRNA under the control of a strong, constitutive promoter. It generates *M. tuberculosis* strains depleted in the desired gene(s) – quicker, efficiently and with high tunability. Another advantage of this system is the ability to silence several genes simultaneously. CRISPRi can be used for investigating the function of *M. tuberculosis* genes, identification of MoA or drug-target validation as was recently disclosed for MmpL3 [57].

### PROSPECT

In 2019, Johnson *et al.* [51] advanced a target-based whole-cell screening approach into a novel screening strategy called PROSPECT (primary screening of strains to prioritize expanded chemistry and targets), which simultaneously identifies whole-cell active compounds and predicts their MoA. Screening of chemical libraries against large *M. tuberculosis* hypomorph pools generated large-scale chemical-genetic interaction profiles (CGIPs), increasing the detection of actives (tenfold more hits) compared with conventional whole-cell screening. Through PROSPECT, 40 new scaffolds against established targets including DNA gyrase, RNAP, cell-wall targets, folate and tryptophan biosynthesis were discovered. PROSPECT also uncovered a new target: the essential efflux pump EfpA. The optimized EfpA inhibitor BRD-8000.3, a methyl-pyrazole derivative of the initial hit, was bactericidal, effective against nonreplicating *M. tuberculosis* and was highly selective for *M. tuberculosis* [51]. This approach has revolutionized not only the screening of compound libraries but also the deconvolution of the MoA as well.

### Targeting nonreplicating and persistent M. tuberculosis

Another crucial point for highly effective TB therapy is targeting phenotypically resistant *M. tuberculosis* that can tolerate antibiotic treatment inside the host without heritable resistance to the first-line anti-TB drugs. Phenotypic tolerance is complex, albeit the literature is replete with various schools of thought on dormancy and/or persistence. Various *in vitro* models of nonreplicating *M. tuberculosis* such as low pH, hypoxia, nutrient starvation, carbon starvation or nitrosative stress are available for whole-cell screening. Each model is significantly different from the others owing to the conditions in which they are established – therefore one needs to be cautious in simplifying screening results. Because one assay is not sufficient to define the nonreplicating stage, recent advances in combining multi-stresses have been important. Gold *et al.* [58] developed a four-stress model that combines acidic pH, mild hypoxia, nitric oxide and butyrate as a carbon source. Importantly, to discriminate between the nonreplicating and replicating active compounds, the charcoal agar resazurin assay (CARA) was developed [59]. Recently, a rapid, low pH, nutrient stress, whole-cell screening assay was developed to facilitate quick determination of the bactericidal activity of test compounds against nonreplicating *M. tuberculosis* [60]. However, the most important question here, in relation to having a nonreplicating *in vivo* infection model, is yet to be answered.

### Caseum assay

It is now widely accepted that, for efficient TB treatment, compounds should not only fulfill the standard pharmacological properties but must reach their specific site of action: tuberculous lesions. Compounds, owing to their physicochemical properties, can display different penetration efficiency inside the cellular and caseous regions of granulomas and cavities (Box 1). To predict this, a rapid equilibrium dialysis assay coupled to liquid chromatography and tandem mass spectrometry was developed to measure the free fraction of compounds in caseum and caseum-like material [61]. The pioneering work of Greenwood *et al.* [62] further underscores the importance of subcellular pharmacokinetics to understand drug efficacy.

### Host-directed therapy

The ‘survival-smartness’ of *M. tuberculosis* is along the basis that it adapts within the early phagosome for replication, avoids fusion with lysosome, which otherwise forms the phagolysosome, counteracts the acidification process and, finally, escapes into the host cytosol. Host-directed therapy (HDT) is an emerging field where compounds are used to modulate the host response(s), thereby reducing the replication or persistence of *M. tuberculosis* and resulting in the efficacy of current and/or new treatments. Compounds are directed at stages of the *M. tuberculosis*-infected macrophage lifecycle to address resistance to *M. tuberculosis* killing. The recent discovery of statins [63] and peptide inhibitors [64] are good examples of HDT in treatment shortening. To fuel HDT research, Huang *et al.* [65] developed a high-throughput assay utilizing cells obtained from *M. tuberculosis*-infected mouse lungs, which they referred to as a deconstructed granuloma platform.

## Concluding remarks

Despite the inherent luxuries of *M. tuberculosis* as a complex pathogen, solid progress has been made in discovering new chemical entities, validating novel drug targets and developing innovative screening tools. However, the development of anti-TB drugs has been held back by inadequate funding. There is a strong rationale that conventional DR-TB treatments should be supplemented with HDTs to achieve reduced tissue damage and improved clinical outcomes. At this juncture, it is noteworthy that, in resource-limited settings specifically in developing countries with high DR-TB burden, there are inherent challenges and barriers to the routine implementation of this strategy of personalized therapy. Nevertheless, to prevent resistance, TB needs to be treated with a combination of several drugs as has been standard practice.
